# ^1^H, ^13^C and ^15^N backbone resonance assignment of BRCA1 fragment 219–504

**DOI:** 10.1007/s12104-020-09963-6

**Published:** 2020-06-24

**Authors:** Michał Górka, Szymon Żerko, Robert Konrat, Wiktor Koźmiński, Dennis Kurzbach

**Affiliations:** 1 Faculty of Chemistry, Biological and Chemical Research Centre, University of Warsaw, Żwirki i Wigury 101, 02-089 Warsaw, Poland; 2 Division of Biophysics, Institute of Experimental Physics, Faculty of Physics, University of Warsaw, Pasteura 5, 02-093 Warsaw, Poland; 3 Department for Structural and Computational Biology, University Vienna, Campus Vienna BioCenter 5, 1030 Vienna, Austria; 4 Faculty of Chemistry, Institute of Biological Chemistry, University Vienna, Währinger Straße 38, 1090 Vienna, Austria

**Keywords:** BRCA1, NMR, Chemical Shift Assignment, IDP, 4D and 5D NMR

## Abstract

The breast cancer susceptibility protein 1 (BRCA1) plays a central role in the suppression of human breast and ovarian cancer. Germ line mutations of the *BRCA1* gene are responsible for the hereditary breast and ovarian cancer (HBOC) syndrome. Here were report ^1^H, ^13^C, and ^15^N resonance assignments for the intrinsically disordered BRCA1 fragment 219–504, which contains important interaction sites for the proto-oncogenic transcription factor MYC as well as for p53. A nuclear magnetic resonance assignment was achieved at 18.8 T magnetic field strength using a 5D HN(CA)CONH experiment and its associated 4D H(NCA)CONH and 4D (H)N(CA)CONH experiments. ^13^C^α^ and ^13^C^β^ assignments were obtained using a 5D HabCabCONH experiment. With this strategy, 90% of ^1^H/^15^N backbone pairs could be assigned. Similarly, 264 C’ resonances were assigned corresponding to 86% of the total number of C’ atoms. In addition, 252 C^β^ resonances (i.e. 85%) were assigned, together with 461 attached H^β^ nuclei, as well as 264 (i.e. 86%) C^α^ resonances, together with 275 attached H^α^ nuclei.

**Electronic supplementary material:**

The online version of this article (10.1007/s12104-020-09963-6) contains supplementary material, which is available to authorized users.

## Biological context

The breast cancer susceptibility protein 1 (BRCA1) is an important factor for human breast and ovarian cancer suppression.(Narod and Foulkes 2004) Indeed, germ line mutations of the *BRCA1* gene have been linked to the hereditary breast and ovarian cancer (HBOC) syndrome in multiple studies. Great efforts have thus been made to unravel the multiple BRCA1-mutation associated tumorigenesis pathways (Castilla et al. 1994; Li et al. 2002; Miki et al. 1994; Narod and Foulkes 2004). Although many details remain elusive, one mechanism is thought to involve disruption of the BRCA1-MYC interaction (Li et al. 2002). The proto-oncogene product MYC forms a transcriptionally active heterodimer with its partner protein MAX (MYC associated factor X). (Conacci-Sorrell et al. 2014; Fieber et al. 2001; Vogt 2012) The MYC/MAX transcription factor network controls fundamental cellular processes such as growth, proliferation, metabolism, and apoptosis. (Tu et al. 2015) In particular, binding of MYC by BRCA1 down-regulates MYC-mediated target gene transcription and, eventually, regulates cell proliferation. (Clark et al. 2012; Wang et al. 1998) Disruption of this regulatory interaction as a result of mutations of BRCA1 is therefore suspected to be involved in HBOC-related tumorigenesis.

The full-length *BRCA1* gene product consists of a stably folded N-terminal RING-domain (residues 1-103) and two C-terminal BRCT domains (residues 1646–1863) connected by a ∼1550 amino acid long intrinsically disordered region (IDR). (Mark et al. 2005) We investigated a construct that consists of residues 219–504 belonging to the central linker domain. This region spans the N-terminal region of the gene product encoded by exon 11, and includes the majority of residues belonging to the reported MYC binding site(s) (residues 433–511) (Wang et al. 1998). Interestingly, this BRCA1 domain contains 11 aromatic amino acids, which is atypical for IDRs. Such residues are prone to intercalation into DNA base stacks, which is consistent with a potential for strong associations of BRCA1 with nucleic acids and its involvement in transcriptional regulation and DNA damage response (Clark et al. 2012).

## Methods and experiments

### Protein expression

A human BRCA1 gene encoding amino acids 219–504 of full-length BRCA1 and an N-terminal histidine tag was sub-cloned into a Pet15b expression vector and transformed into *E.coli* Rosetta pLysS cells. Cells were grown at 37 °C in M9 medium containing 1 g/L ^15^N ammonium chloride and 3 g/L ^13^C glucose for ^13^C and ^15^N labeling. Cell cultures were induced at an optical absorption (A_600 nm_) of 0.8 using 0.5 mM IPTG (Isopropyl β-d-1-thiogalactopyranoside). After 3 h incubation at 37 °C, the cells were harvested, and the resulting cell pellets were lysed by sonication in 25 mM TRIS (tris(hydroxymethyl)aminomethane) buffer at pH 8 containing 100 mM NaCl and 1 mM β-mercaptoethanol. The cell lysate was cleared by centrifugation at 18 000 rpm for 20 min. For protein purification, the supernatant was heated to 90 °C for 5 min, and the resulting precipitate was removed through centrifugation again at 18 000 rpm for 20 min.

The supernatant containing the soluble protein fraction was loaded onto a Ni^2+^-loaded HiTrap 5 ml affinity column (GE Healthcare) for purification and eluted by a buffer containing 20 mM TRIS at pH 8, 50 mM NaCl, and 0.5 M imidazole using a linear gradient of 15 column volumes.

The final product was dialyzed into a buffer containing 25 mM MES, and 25 mM NaCl (pH 5.5) in a 90% H_2_O/10% D_2_O mixture. To prevent precipitation, 100 mM arginine hydrochloride were added. The final protein concentration was 0.3 mM.

### NMR spectroscopy

^1^H, ^13^C, and ^15^N resonance assignments were achieved with a combination of high-dimensionality experiments and sparse random sampling of indirectly detected time domains. This combination enables sufficient resolution for assignment of intrinsically disordered domains that contain ca. 300 amino acids, while permitting data acquisition within feasible experimental times. A 3D HNCO experiment was used to obtain a reference spectrum for sparse multidimensional Fourier transform (SMFT) processing of the higher dimensionality experiments. (Kazimierczuk et al. 2009)

**Fig. 1:**
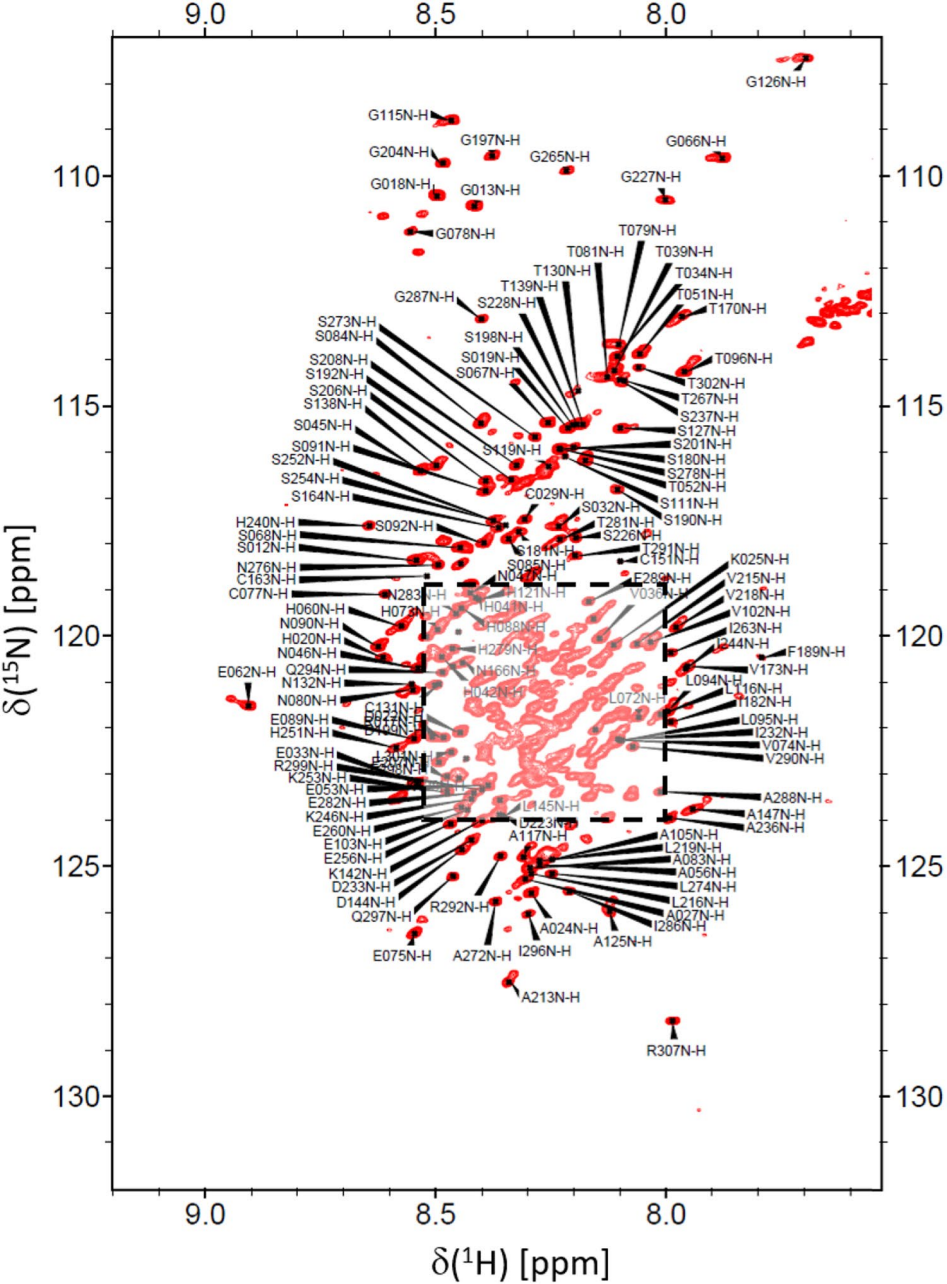
^1^H-^15^N HSQC spectrum of BRCA1 fragment 219–503 obtained at 800 MHz and a temperature of *T* = 298 K. The resonance assignments are indicated

**Fig. 2:**
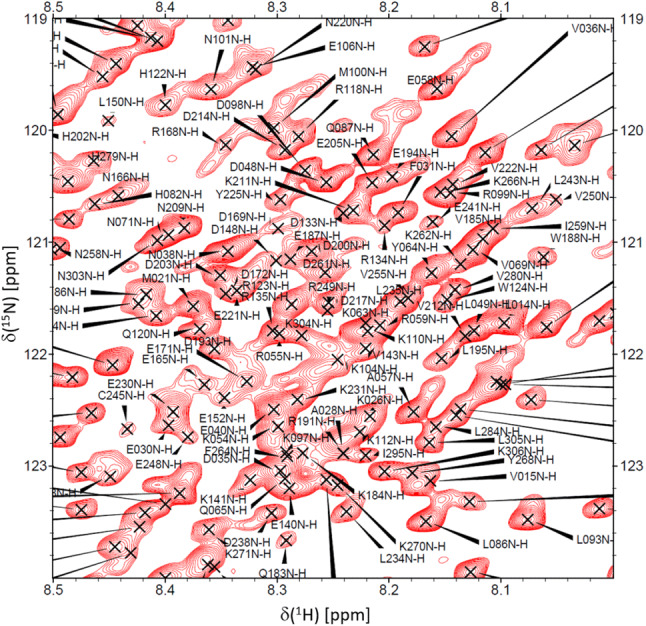
Close-up view of the region indicated by the dashed box in Fig. 1

Backbone resonance assignment was achieved using a 5D HN(CA)CONH (Kazimierczuk et al. 2010) experiment and its associated 4D H(NCA)CONH and 4D (H)N(CA)CONH experiments. ^13^C^α^ and ^13^C^β^ assignments were obtained using the 5D HabCabCONH^16^ experiment. All experiments were acquired at 298 K on an Agilent DirectDrive 2 800 MHz spectrometer equipped with a cryogenically cooled 5 mm ^1^H–^13^C–^15^N triple-resonance probe. 3D, 4D and 5D experiments were sparsely sampled using on-grid sampling schedules randomly drawn from a truncated Gaussian distribution with standard deviation set to half of the maximal evolution time (see the supplementary material for the sampling parameters). All NMR data sets were processed by multidimensional Fourier transformations using home written software packages. (Kazimierczuk et al. 2009; Kazimierczuk et al. 2006; Stanek et al. 2012; Stanek and Kozminski 2010) Sampling artefacts from the 3D HNCO as well as from the 4D and 5D experiments were removed using the Signal Separation Algorithm (Stanek and Kozminski 2010), as implemented in the ‘cleaner3d’ (Stanek and Kozminski 2010),‘cleaner4d’ (Stanek et al. 2012), and ‘cleaner5d’ (Kosiński et al. 2017) programs, respectively. The resonance assignment was supported by the TSAR program. (Zawadzka-Kazimierczuk et al. 2012) Input peak lists were prepared by manual peak-picking using the Sparky software package (Goddard and Kneller).

## Extent of the assignment and data deposition

**Fig. 3:**
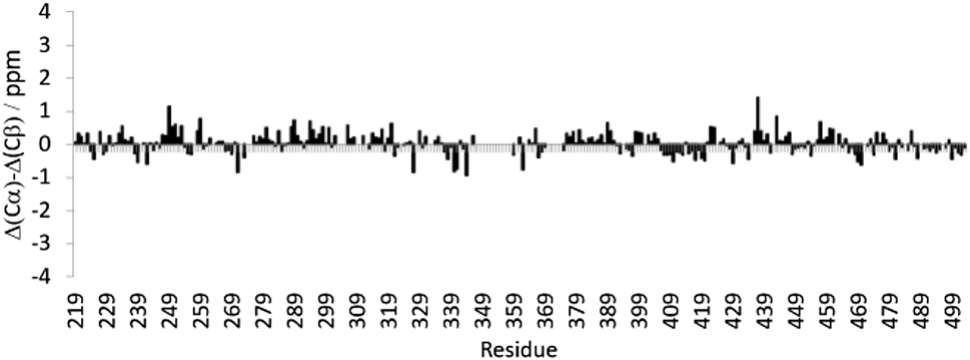
Secondary ^13^C chemical shift analysis of BRCA1 fragment 219–504. The small observed deviations from predicted random-coil chemical shifts corroborate data that suggest that the central linker domain of BRCA1 remains intrinsically disordered in solution

The ^1^H-^15^N HSQC spectrum of BRCA1 fragment 219–504 shown in Figs. 1 and 2 displays a rather narrow chemical shift dispersion in the ^1^H dimension, indicating that the assigned fragment is unlikely to adopt a stable secondary structure. This is in agreement with prior studies of BRCA1, (Mark et al. 2005) that showed that the central linker domain of this protein is indeed best characterized as an intrinsically disordered region (IDR). This is corroborated by a secondary ^13^C chemical shift analysis (Fig. 3) that shows only minor deviations, Δ(C^α^) and Δ(C^β^), from predicted, neighborhood-corrected random-coil chemical shifts (expressed in Fig. 3 as Δ(C^α^) - Δ(C^β^) = (C^α^_experimental_ − C^α^_predicted_) − (C^β^_experimental_ − C^β^_predicted_)).(Tamiola and Mulder 2012) Using the methods outlined in the previous section, 90% (264 out of 294) of ^1^H/^15^N backbone pairs could be assigned. Similarly, 264 C’ resonances were assigned corresponding to 86% of the total number.

In addition, 252 C^β^ resonances (i.e. 85%) were assigned, together with 461 attached H^β^ nuclei, and 264 (i.e. 86%) C^α^ resonances were assigned, together with 275 attached H^α^ nuclei.

The ^1^H, ^13^C, and ^15^N chemical shifts have been deposited in the biological magnetic resonance data bank (BioMagResBank) under the BMRB submission number 50231 (http://www.bmrb.wisc.edu/).

**Table:** 

Experiment dimension	HNCO		(H)N(CA)CONH			(HN)CO(NCA)CONH		
	N	CO	N	CO	N	CO	CO	N
Maximum evolution time (ms)	75	75	28	20	20	15	7	20
Spectral width (kHz)	2.5	2.5	2.5	2.5	2.5	2.5	2.5	2.5
Number of points	1520		6000			3600		
Experiment duration (h)	8.2		63.5			39.9		
Sampling density versus conventional	4.32 × 10^− 2^		2.45 × 10^− 2^			2.06 × 10^− 2^		

Experiment dimension	HN(CA)CONH				HabCabCONH			
	H	N	CO	N	HAHB	CACB	CO	N
Maximum evolution time (ms)	20	28	20	28	15	7	20	28
Spectral width (kHz)	2.8	2.5	2.5	2.5	4	12	2.5	2.5
Number of points	2800				2500			
Experiment duration (h)	61.7				61.9			
Sampling density versus conventional	2.29 × 10^− 4^				2.10 × 10^− 4^			

## Electronic supplementary material

Below is the link to the electronic supplementary material.

Supplementary file 1 (TXT 57 kb)

## Data Availability

The ^1^H, ^13^C, and ^15^N chemical shifts have been deposited in the biological magnetic resonance data bank (BioMagResBank) under the BMRB access number 50231 (http://www.bmrb.wisc.edu/). The data leading to the assignment can be obtained from the authors upon request.

## Code Availability

The sparky software(Goddard and Kneller) that was used for resonance assignment can be obtained from https://www.cgl.ucsf.edu/home/sparky/. The TSAR program (Zawadzka-Kazimierczuk et al. 2012) can be obtained from http://nmr.cent3.uw.edu.pl/software/category/6-tsar-tool-for-smft-based-assigment-of-resonances.
